# Nitric Oxide Detoxification by *Mesorhizobium loti* Affects Root Nodule Symbiosis with *Lotus japonicus*

**DOI:** 10.1264/jsme2.ME21038

**Published:** 2021-08-31

**Authors:** Mitsutaka Fukudome, Yuta Shimokawa, Shun Hashimoto, Yusuke Maesako, Nahoko Uchi-Fukudome, Kota Niihara, Ken-ichi Osuki, Toshiki Uchiumi

**Affiliations:** 1 Graduate School of Science and Engineering, Kagoshima University, 1–21–35 Korimoto, Kagoshima 890–0065, Japan; 2 Division of Symbiotic Systems, National Institute for Basic Biology, 38 Nishigonaka, Myodaiji, Okazaki, Aichi 444–8585, Japan; 3 Graduate School of Medical and Dental Sciences, Kagoshima University, 8–35–1 Sakuragaoka, Kagoshima 890–8544, Japan

**Keywords:** hemoglobin, flavohemoglobin, nitric oxide, root nodule symbiosis

## Abstract

Root nodule symbiosis between legumes and rhizobia involves nitric oxide (NO) regulation by both the host plant and symbiotic rhizobia. However, the mechanisms by which the rhizobial control of NO affects root nodule symbiosis in *Lotus japonicus* are unknown. Therefore, we herein investigated the effects of enhanced NO removal by *Mesorhizobium loti* on symbiosis with *L. japonicus*. The *hmp* gene, which in *Sinorhizobium meliloti* encodes a flavohemoglobin involved in NO detoxification, was introduced into *M. loti* to generate a transconjugant with enhanced NO removal. The symbiotic phenotype of the transconjugant with *L. japonicus* was examined. The transconjugant showed delayed infection and higher nitrogenase activity in mature nodules than the wild type, whereas nodule senescence was normal. This result is in contrast to previous findings showing that enhanced NO removal in *L. japonicus* by class 1 phytoglobin affected nodule senescence. To evaluate differences in NO detoxification between *M. loti* and *L. japonicus*, NO localization in nodules was investigated. The enhanced expression of *class 1* *phytoglobin* in *L. japonicus* reduced the amount of NO not only in infected cells, but also in vascular bundles, whereas that of *hmp* in *M. loti* reduced the amount of NO in infected cells only. This difference suggests that NO detoxification by *M. loti* exerts different effects in symbiosis than that by *L. japonicus*.

Nitric oxide (NO) is produced in plants as a biotic or abiotic response (Delledonne *et al.*, 1998; Beligni and Lamattina, 2000; Dordas *et al.*, 2003a, 2003b; Qiao and Fan, 2008; Simontacchi *et al.*, 2015). Previous studies that focused on the production and detoxification of NO in plant–microbe interactions reported that NO was detectable in both pathogenic and symbiotic responses and was widely involved in these interactions (Romero-Puertas *et al.*, 2004; Wendehenne *et al.*, 2004; Delledonne, 2005; Baudouin *et al.*, 2006; Pii *et al.*, 2007; Leitner *et al.*, 2009; Gaupels *et al.*, 2011; Murakami *et al.*, 2011). In root nodule symbiosis (RNS) between legumes and rhizobia, NO has been detected at various stages from infection to nodule senescence, indicating the importance of NO regulation for the establishment of RNS (Baudouin *et al.*, 2006; Nagata *et al.*, 2008; del Giudice *et al.*, 2011; Cam *et al.*, 2012; Hichri *et al.*, 2015; 2016; Meilhoc *et al.*, 2015; Fukudome *et al.*, 2016). NO is controlled by hemoglobin derived from both the host plant and rhizobia (Meilhoc *et al.*, 2010; Fukudome *et al.*, 2016; 2019a; Berger *et al.*, 2020; Larrainzar *et al.*, 2020; Salas *et al.*, 2020). Host plants remove NO by plant hemoglobin, now generally known as phytoglobin, specifically by non-symbiotic class 1 phytoglobin (Phytogb1) (Hill *et al.*, 2016; Becana *et al.*, 2020; Larrainzar *et al.*, 2020). In *Lotus japonicus*, the low expression of the *LjGlb1-1* gene, which encodes Phytogb1, reduces infection efficiency and symbiotic nitrogen fixation (SNF) (Fukudome *et al.*, 2016). SNF increases in hairy root cultures and transgenic lines that highly express *LjGlb1-1*, and nodule senescence is also delayed in these transgenic lines (Shimoda *et al.*, 2009; Fukudome *et al.*, 2019a). Similarly, the low expression of the Phytogb1-encoding *Glb1.1* gene in *Medicago truncatula* decreased SNF, while high expression increased it (Berger *et al.*, 2020).

Symbiotic rhizobia remove NO by the flavohemoglobin Hmp, such as in *Sinorhizobium meliloti* (Meilhoc *et al.*, 2010; del Giudice *et al.*, 2011; Cam *et al.*, 2012), or by a single-domain globin, such as Bjgb of *Bradyrhizobium diazoefficiens* (Cabrera *et al.*, 2011; 2016; Sánchez *et al.*, 2011). In a strain of *S. meliloti* deficient in the *hmp* gene, infected nodules showed low SNF and early senescence (Cam *et al.*, 2012; Blanquet *et al.*, 2015), whereas in a strain that highly expresses *hmp*, nodules in the late stage of RNS showed higher SNF and delayed senescence than those infected with wild-type (WT) bacteria (Cam *et al.*, 2012). The *hmp* and *bjgb* genes both contribute to NO tolerance in rhizobia under free-living conditions (Meilhoc *et al.*, 2010; Cabrera *et al.*, 2011; 2016; Sánchez *et al.*, 2011), and NO reduction by NorB and NorC in the denitrification pathway and NO metabolism by NnrS1 and NnrS2 have been reported (Cabrera *et al.*, 2011; 2016; Meilhoc *et al.*, 2013; Blanquet *et al.*, 2015). Although NO functions as a signaling molecule in diverse physiological responses (Delledonne *et al.*, 1998; Beligni and Lamattina, 2000; Pagnussat *et al.*, 2002; He *et al.*, 2004; Qiao and Fan, 2008; Mur *et al.*, 2013), it also inhibits nitrogenase activity (Trinchant and Rigaud, 1982; Kato *et al.*, 2010); therefore, these regulatory mechanisms are extremely complex, which makes it difficult to understand the full extent of NO regulation in each symbiosis.

NO regulation in RNS has generally been investigated using *L. japonicus*, *M. truncatula*, and *Glycine max* as host plants or in symbiosis between non-leguminous actinorhizal plants and *Frankia* (Sasakura *et al.*, 2006; Niemann and Tisa, 2008; Coats *et al.*, 2009). Specifically, the effects of deficient or enhanced NO regulation by symbiotic rhizobia have been examined in the RNS of *M. truncatula* infected with *S. meliloti*, but not *L. japonicus*. However, we previously reported that deficient or enhanced NO detoxification by *LjGlb1-1* (NO detoxification derived from the host plant) affected the RNS of *L. japonicus* with *Mesorhizobium loti* (Shimoda *et al.*, 2009; Fukudome *et al.*, 2016; 2019a; 2019b). Since the developmental process and morphology of *L. japonicus* root nodules differ from those of *M. truncatula* (Hirsch, 1992; Larrainzar *et al.*, 2020), the difference in symbiotic nodule organogenesis is often described as one of the factors causing variations in the role and control of NO; however, the underlying mechanisms remain unclear (del Giudice *et al.*, 2011; Hichri *et al.*, 2015; Fukudome *et al.*, 2016). Although a more detailed understanding of the contributions that *L. japonicus* and *M. loti *make to the control of NO during RNS is needed in order to discuss differences and similarities in the roles and regulation of NO, research into this issue has been difficult. Some genes in the *Mesorhizobium* species are annotated as encoding flavohemoproteins (Larrainzar *et al.*, 2020); however, the homology of these genes to *hmp* in the more-researched *S. meliloti* is low and their NO-scavenging activity and NO responsiveness are unknown. Furthermore, genes known to be involved in NO metabolism, *bjgb* and *norBC*, have not been identified in the genome of *M. loti* MAFF303099; therefore, the mechanisms underlying NO metabolism in *M. loti*, which is symbiotic with *L. japonicus*, have not yet been elucidated.

The present study investigated the effects of enhanced NO detoxification by symbiotic rhizobia in the RNS of *L. japonicus*–*M. loti*. A transconjugant of *M. loti* that ectopically expressed the *hmp* gene of *S. meliloti* was generated and its symbiotic phenotype was characterized based on comparisons of the infection process, resistance to NO, and nodular senescence in the Hmp-expressing strain with those of a WT strain. Unexpected results in senescence prompted us to investigate the localization of NO in nodules.

## Materials and Methods

### Growth conditions of plants

Plants of *L. japonicus* accession Gifu B-129 and its derivative lines were germinated and grown as previously described (Nagata *et al.*, 2008). In brief, 5‍ ‍d after germination, seedlings were transferred to 1.5% Fåhraeus agar plates (Fåhraeus, 1957) and inoculated with a cell suspension (10^7^‍ ‍cells‍ ‍mL^–1^ in water) of *M. loti* MAFF303099 (Kaneko *et al.*, 2000) and its derivative strains. Plants were grown under controlled conditions with photosynthetically active radiation of 150‍ ‍μmol photons m^–2^ s^–1^ (16-h photoperiod) at 25°C for up to 8‍ ‍weeks post-inoculation (wpi). The *LjGlb1-1* overexpression line (Ox1) of *L. japonicus* was produced according to the method reported by Aoki *et al.* (2002) using a binary vector with the CaMV 35S promoter and cDNA of *LjGlb1-1* that was constructed based on pIG121-Hm. The Ox1 line was used in the same manner as that generated by Fukudome *et al.* (2019a; 2019b).

### Gene editing and cloning of *M. loti* strains

Since *M. loti* MAFF303099 did not stably maintain the plasmid pBBR-*hmp* (Meilhoc *et al.*, 2010) during RNS (data not shown), we integrated the *hmp* gene containing the promoter sequence into the genome of *M. loti* MAFF303099. Using pBBR-*hmp* as a template, the DNA region that codes from the *hmp* gene to the gentamicin resistance gene (*hmp*–Gm^r^, approximately 2.7‍ ‍kb) was amplified by PCR using the primer pair of 5′-cgcggccttggcctggaacccctagaagc-3′ and 5′-ccatcttctcgcttcacaga-3′. On the genome of *M. loti* MAFF303099, a region that does not affect growth or RNS and that ranges between approximately 1‍ ‍kb upstream and downstream of the *mlr8031* gene locus (*mlr8031*-UP and *mlr8031*-DW) was amplified by PCR. The primer pair of 5′-ttacctgcaggcatagcctcggcgggt-3′ and 5′-cggcggccttgcctgcctggaacccctagaagctgtctttttatc-3′ and that of 5′-cggcggcctttccctctctcactcggcaaacag-3′ and 5′-atcctctctagaaaacccaaag-3′ were used for up- and downstream PCR, respectively. The DNA fragments of *mlr8031*-UP, *hmp*–Gm^r^, and *mlr8031*-DW were linked by crossover PCR. The resulting fragment (*mlr8031*-UP/*hmp*–Gm^r^/*mlr8031*-DW) was cloned into the suicide plasmid pK18*mobsacB*, which retains the SacB marker, with the aid of the restriction enzymes *Sse*8387I and *Xba*I (both from Takara Bio). The resulting plasmid (pK18*mobsacB*/*mlr8031*-UP/*hmp*–Gm^r^/*mlr8031*-DW) was transformed into *Escherichia coli* HST08 (Takara Bio) and then transferred into *M. loti* and DsRed-labeled *M. loti* (*M. loti*-DsRed, Maekawa *et al.*, 2009) by tri-parental mating using helper *E. coli* MM294 (pRK2013). To obtain transconjugants derived from *M. loti* and *M. loti*-DsRed with the plasmid pK18mobsacB/*mlr8031*-UP/*hmp*–Gm^r^/*mlr8031*-DW integrated into their genomes by homologous recombination, the resulting strains were spread on TY agar plates containing gentamicin (50‍ ‍μg mL^–1^) or kanamycin (50‍ ‍μg mL^–1^), respectively. Colonies were screened by sensitivity to 10% sucrose, and two sensitive clones in which the *hmp* gene was amplified by PCR were referred to as W315 (derived from *M. loti*) and RW45 (derived from *M. loti*-DsRed). To confirm that *mlr8031* was replaced by homologous recombination with *hmp*–Gm^r^, W315 was spread on TY agar medium containing 10% sucrose and gentamicin (50‍ ‍μg mL^–1^) and colonies were subjected to PCR. One of the clones was selected and referred to as transconjugant E109. The bacterial strains and plasmids used in the present study are listed in Table 1. To confirm *hmp* expression, we used the primer set 5′-tgcttgcgtctatcaaggag-3′ and 5′-ggttcttctcacggacgatg-3′ for *hmp*, and the primer set 5′-gccctctgctcgacctttcc-3′ and 5′-agcatcgccatcgtgtcctc-3′ for *sigA* as housekeeping genes. All bacterial strains were cultured in liquid HM medium (Cole and Elkan, 1973).

### Bacterial growth conditions and the NO resistance assay

When the OD_600_ of the culture reached between 0.4 to 0.5, cells were harvested and washed twice with HM medium. Cells were then suspended in HM medium to achieve an OD_600_ equal to 0.2. The NO donor, sodium nitroprusside (SNP), was added to the bacterial suspension to final concentrations of 5, 25, 50, 100, 250, and 500‍ ‍μM, and following by an incubation with shaking at 26°C for 12 h. OD_600_ was measured using Miniphoto518R (Taitec) every 2 h during the incubation. As a control, potassium ferricyanide (PF) was used at the same concentrations as SNP.

### Nitrogenase activity

The nitrogenase activity of nodules was assessed by measuring acetylene reduction activity (ARA) according to the method reported by Shimoda *et al.* (2009). Whole plants were placed in glass tubes containing wet filter paper. The tubes were filled with an acetylene and air mixture (C_2_H_2_:air=1:9‍ ‍[v/v]). After a 1-h incubation at 25°C, the amount of ethylene in the gas phase was evaluated using a GC-3A gas chromatograph (Shimadzu).

### Endogenous NO production in and NO released from nodules

The endogenous production of NO in nodules at 4 and 6‍ ‍wpi was monitored by fluorescence microscopy, as described by Fukudome *et al.* (2020). The probes were dissolved in phosphate-buffered saline: 137‍ ‍mM NaCl, 2.7‍ ‍mM KCl, 8‍ ‍mM Na_2_HPO_4_, and 2‍ ‍mM NaH_2_PO_4_ (pH 7.4). To detect NO inside cells, 5% agar sections of nodules were soaked for 1 h in 20‍ ‍μM diaminofluorescein-FM diacetate (DAF-FM DA) (Goryo Chemical). DAF-FM DA has membrane permeability and is deacetylated to DAF-FM by esterase inside cells, upon which DAF-FM reacts with the endogenous NO oxidation product N_2_O_3_ to form a highly fluorescent triazole. The nodule cell walls in sections were stained for 15‍ ‍min with calcofluor white stain (Sigma-Aldrich). Fluorescence images were captured with an A1si-90i microscope and epifluorescence images with an Eclipse 90i microscope (both from Nikon). Fluorescence intensity was quantified using the image analysis software ImageJ (Version 1.51; NIH, Bethesda, MD, USA). Images (encoded in the 16-bit mode with 65,536 greyscale values) of infected cells were selected at random for each line, and the average fluorescence intensity of the region was measured (*n*=30). NO released from nodules was assessed using the cell-impermeant probe DAF-FM. Detached nodules were immediately soaked in 7‍ ‍μM DAF-FM for 15‍ ‍min. The fluorescence of DAF-FM solution was measured using a Qubit 3.0 fluorometer (Thermo Fisher Scientific) with excitation at 430–495‍ ‍nm and emission at 510–580‍ ‍nm.

## Results

### NO resistance in the *Hmp*-expressing strain

**Fig. 1.**
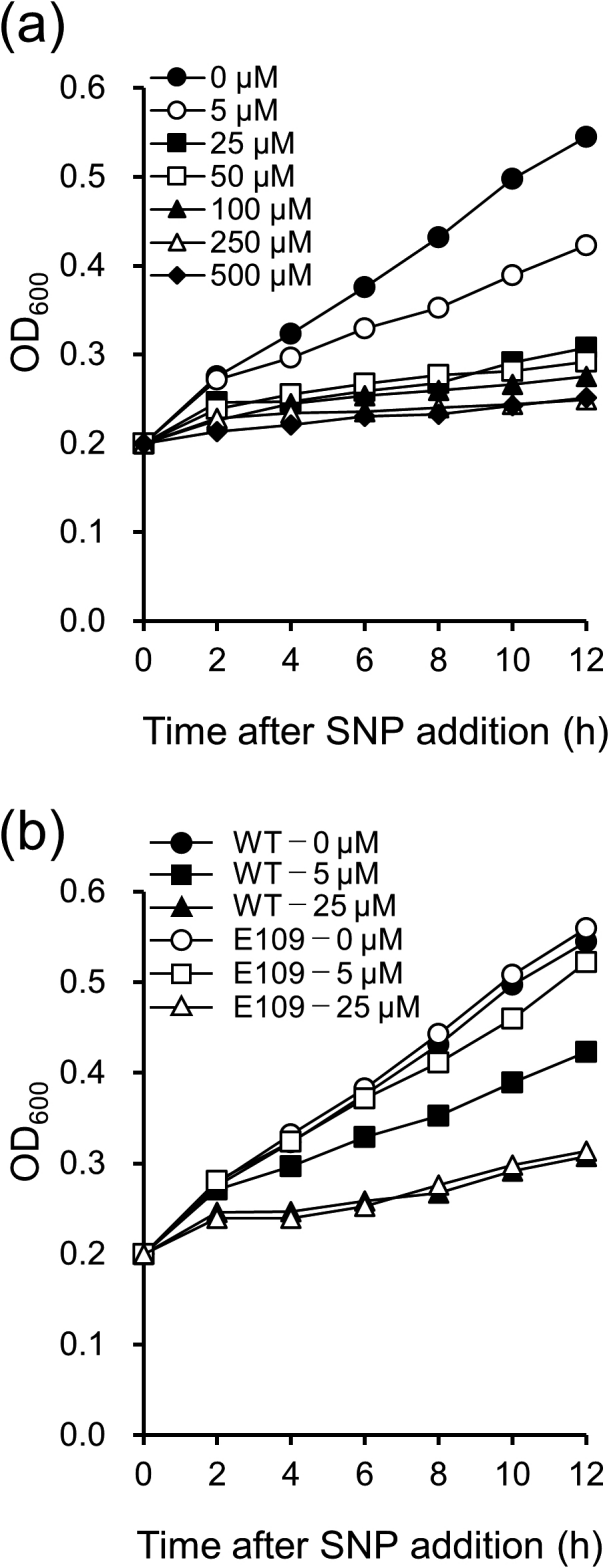
Effects of the *hmp* gene from *Sinorhizobium meliloti* on the growth of *Mesorhizobium loti* strains. (a) *M. loti* WT cells were cultured with or without various concentrations of SNP, as indicated. (b) *M. loti* WT (black symbols) or E109 cells (open symbols) were cultured with (5‍ ‍μM or 25‍ ‍μM) or without SNP. Cell growth was measured every 2 h after the addition of SNP. In (a) and (b), values are the mean of four biological replicates.

Resistance to NO under free-living conditions was compared between WT *M. loti* MAFF303099 and the Hmp-expressing transconjugant E109. The transconjugant E109 was confirmed to express the *hmp* gene by RT-PCR (Supplementary Fig. S1), and the growth of WT in the presence of SNP as a NO donor was then measured (Fig. 1a). SNP at 5‍ ‍μM inhibited the growth of WT, with higher concentrations of SNP (>25‍ ‍μM) strongly inhibiting growth. WT and E109 were cultured in the presence of 0, 5, and 25‍ ‍μM SNP (Fig. 1b). Under control conditions (0‍ ‍μM), no significant difference was detected in the growth of WT and E109. At 5‍ ‍μM SNP, the growth of WT was significantly inhibited, while that of E109 was suppressed to a lesser extent. At 25‍ ‍μM SNP, the growth of both strains was significantly inhibited. To rule out the possibility that growth was inhibited by cytotoxicity independent of NO from SNP, we also examined the effects of 5‍ ‍μM PF on the growth of WT and E109 (Supplementary Fig. S2). PF at 5‍ ‍μM did not inhibit the growth of WT or E109.

### Infection and nodulation by *Hmp*-expressing strains

**Fig. 2.**
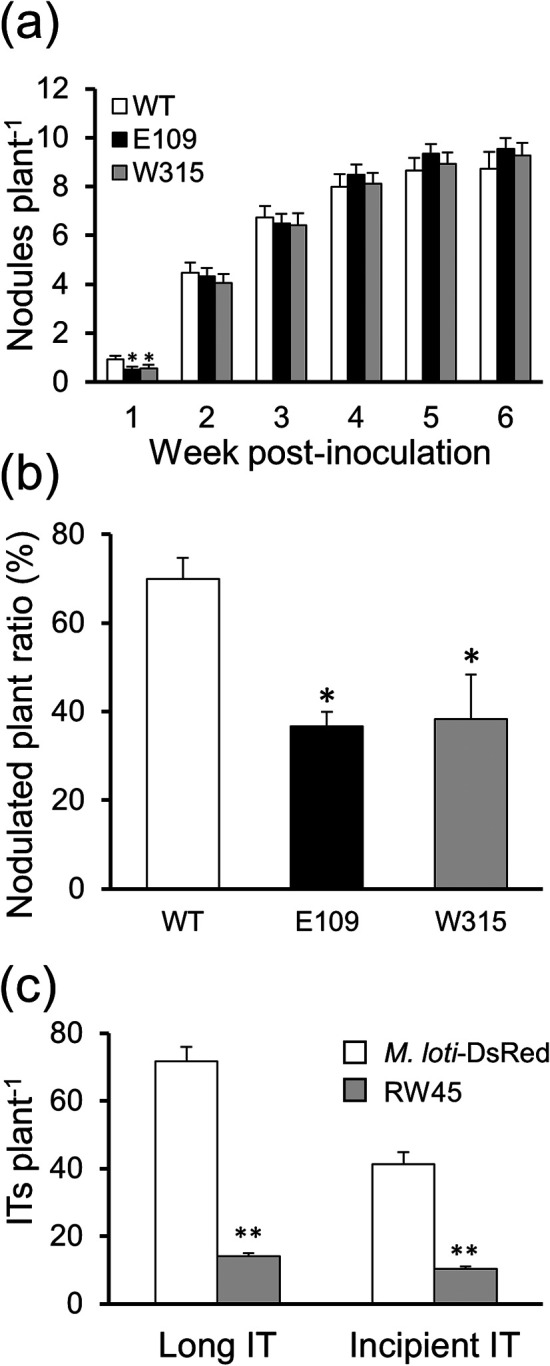
Nodulation and formation of infection threads. (a) The number of nodules per plant was counted at 1–6 wpi. Values indicate the mean±SE (*n*=40). (b) The ratio of plants with nodulation was assessed at 1 wpi. Values indicate the mean±SE (*n*=40). (c) The number of infection threads per plant was counted at 10 dpi. Values indicate the mean±SE (*n*=35). Asterisks in (a), (b), and (c) denote significant differences from the WT or *M. loti*-DsRed strain (the Student’s *t*-test, * *P*<0.05, ** P<0.01).

The number of nodules that formed on *L. japonicus* from 1 to 6 wpi was compared in the strains E109, W315, and WT (Fig. 2a). Although no significant differences were observed in the number of nodules between 2 and 6 wpi, E109 and W315 induced fewer nodules than WT at 1 wpi and the number of plants that formed nodules by 1 wpi was also lower (Fig. 2b). The number of infection threads (ITs) formed by DsRed-labeled WT (*M. loti*-DsRed) and RW45 were counted at 10‍ ‍d post-inoculation (dpi) (Fig. 2c). ITs were categorized into two groups, incipient or long ITs, according to the terminology of Małolepszy *et al.* (2015), except that we included elongating ITs in the long IT category. The numbers of long and incipient ITs were both significantly smaller in RW45 than in *M. loti*-DsRed. The fresh weights of plants and total nodules from 3 to 8 wpi did not significantly differ between E109 and WT (Supplementary Fig. S3).

### Symbiotic phenotypes of mature nodules formed by an *Hmp*-expressing transconjugant

**Fig. 3.**
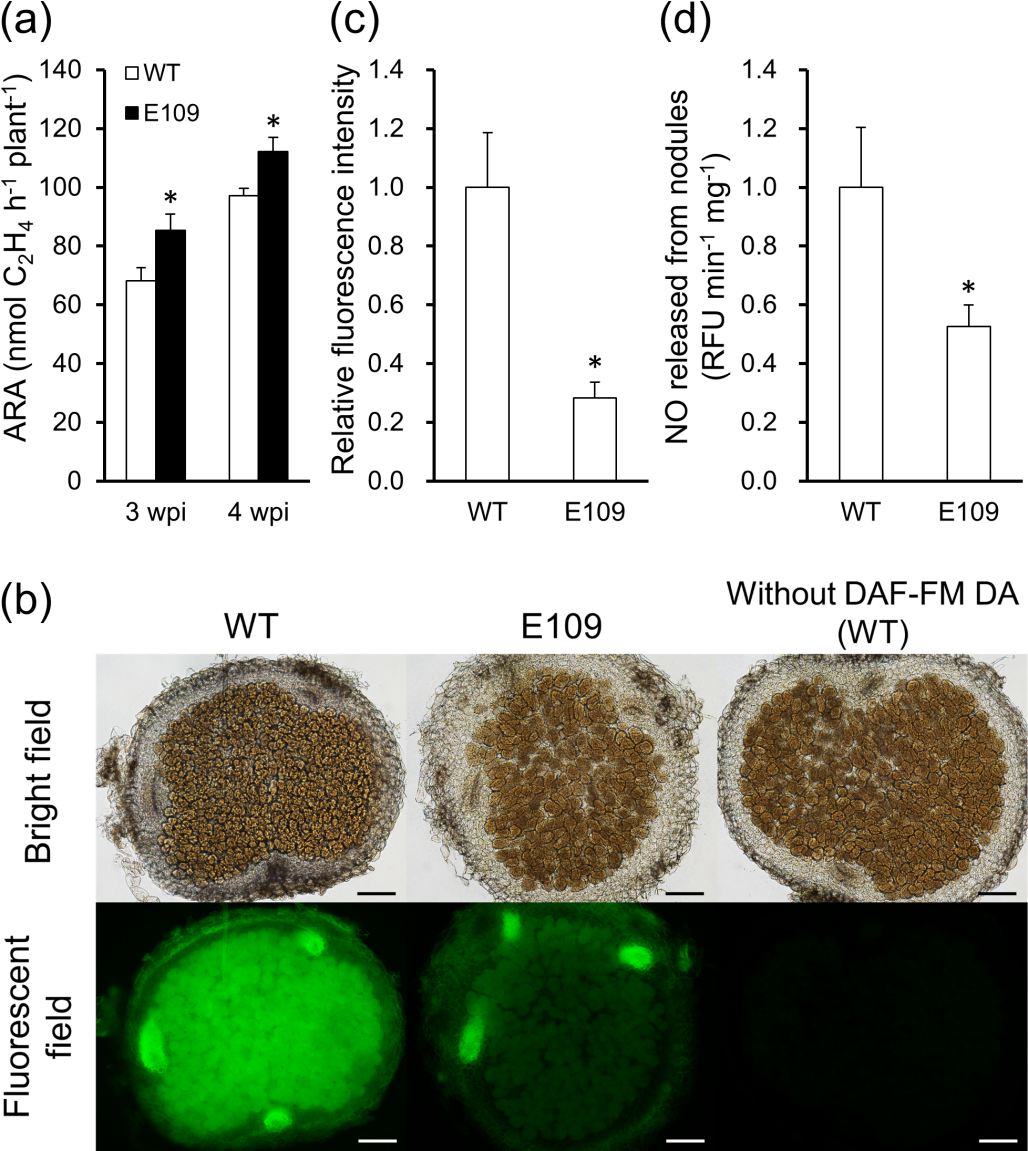
Nitrogenase activity and NO levels in mature nodules. (a) Nitrogenase activity (measured as ARA) was assessed at 3 and 4 wpi (mature nodules). Values indicate the mean±SE (*n*=24). (b) Fluorescence microscopy images were taken of agar sections of nodules at 4 wpi incubated with the fluorescent probe DAF-FM DA (sample images shown). Scale bars, 100‍ ‍μm. (c) The fluorescence intensity of each image taken at 4 wpi was quantified using ImageJ. Values indicate the mean±SE (*n*=40). (d) NO released from nodules at 4 wpi was measured as fluorescence intensity with the DAF-FM probe and quantified. Values indicate the mean±SE (*n*=12). In (c) and (d), fluorescence intensity in E109 is expressed relative to that in WT, which was set at 1. Asterisks denote significant differences from WT (the Student’s *t*-test, * *P*<0.05).

The symbiotic phenotype of mature nodules was compared at 3 and 4 wpi (Fig. 3). To assess nodule function, ARA was measured to evaluate nitrogenase activity, which was found to be significantly higher in mature nodules with E109 than in those with WT at both 3 and 4 wpi (Fig. 3a). To assess the ability of E109 to scavenge NO in nodules, the fluorescence intensities from the NO-specific probes DAF-FM DA and DAF-FM were measured (Fig. 3b, c, and d). After labeling with DAF-FM DA to reveal endogenous NO levels in nodules, cells in mature nodules infected with E109 exhibited lower levels of intrinsic NO than those with WT (Fig. 3b). A comparison using ImageJ also supported significantly lower NO levels in E109 than in WT (Fig. 3c). NO released from nodules was revealed by labeling with DAF-FM, and the results obtained showed that NO-dependent fluorescence from released NO in mature nodules was lower in E109 than in WT (Fig. 3d).

### Symbiotic phenotype of senescent nodules in *Hmp*-expressing strains

**Fig. 4.**
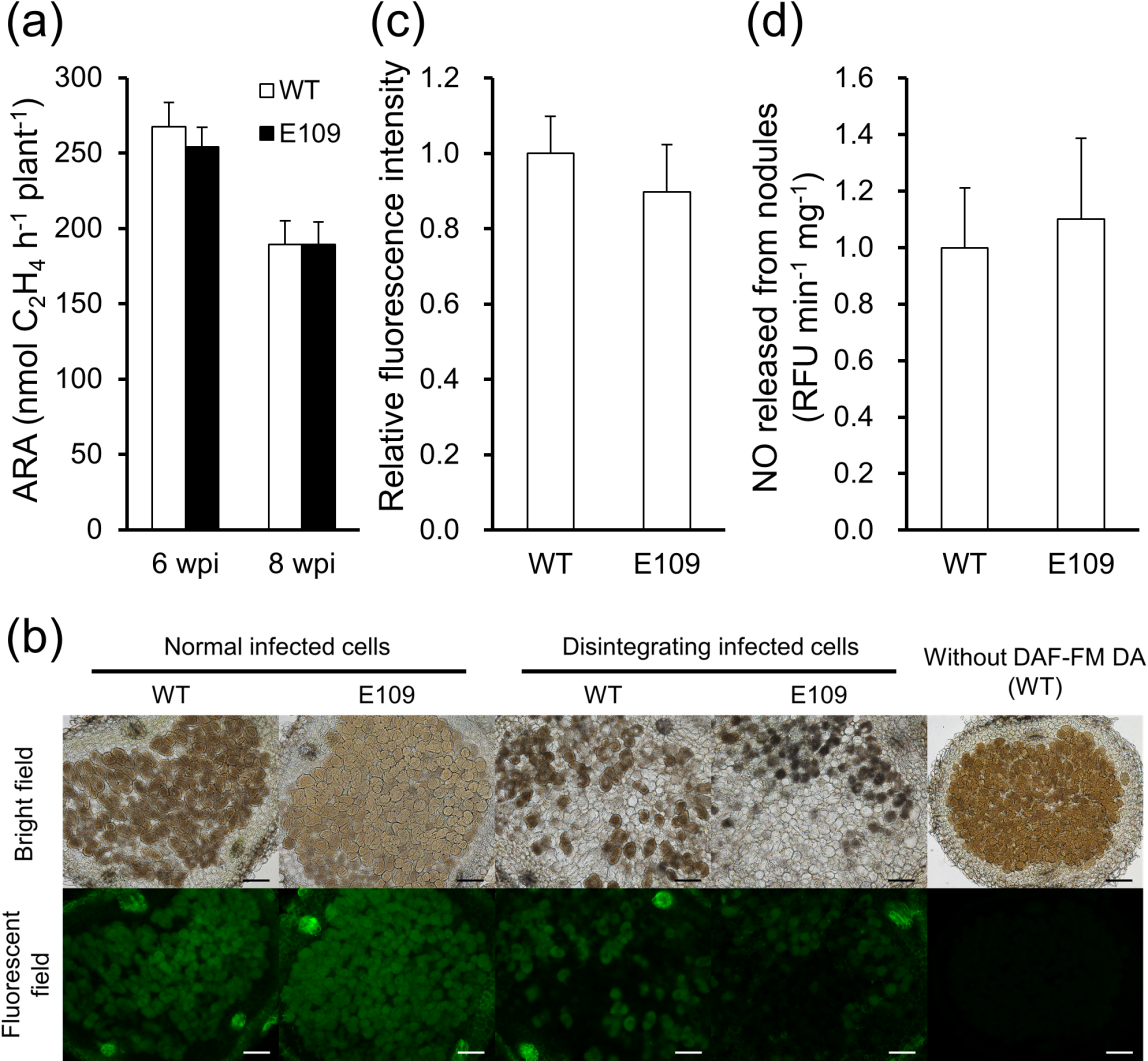
Nitrogenase activity and NO levels in senescent nodules. (a) Nitrogenase activity (estimated as ARA) was measured at 6 and 8 wpi (late stage of symbiosis). Values indicate the mean±SE (*n*=40). (b) Fluorescence microscopy images were taken of senescent nodules incubated with DAF-FM DA. Scale bars, 100‍ ‍μm. (c) The fluorescence intensity of each image taken of senescent nodules was quantified using ImageJ. Values indicate the mean±SE (*n*=40). (d) NO released from senescent nodules was assessed by measuring fluorescence intensity at 6 wpi (4‍ ‍weeks after nodulation). Fluorescence was quantified and expressed per min and per weight of fresh nodules. Values indicate the mean±SE (*n*=12). In (c) and (d), the fluorescence intensity of E109 is expressed relative to that of WT, which was set at 1. In (a), (c), and (d), none of the values showed significant differences (the Student’s *t*-test, *P*<0.05).

The phenotypes of old nodules in plants infected with WT and E109 were compared at 6 and 8 wpi (Fig. 4). No significant differences were observed in overall nitrogenase activity per plant at 6 or 8 wpi (Fig. 4a). To examine nitrogenase activity specifically in old nodules, we measured ARA at 6 wpi in nodules that had appeared at 2 wpi and found that nitrogenase activity in these nodules did not significantly differ between E109 and WT (Supplementary Fig. S4a). Neither endogenous nor released NO in old nodules significantly differed between WT and E109 (Fig. 4b, c, and d). Normal and disintegrating infected cells were observed in old nodules, and the progression of aging in nodules induced by WT and by E109 was similar (Fig. 4b). Furthermore, no significant differences were noted in the rate of greening in old nodules, indicating similar advances in senescence in nodules induced by WT or E109 (Supplementary Fig. S4b). In all experiments on late-stage symbiosis, no significant differences were observed between E109 and WT.

### Differences in effects of *Hmp* and phytoglobin on NO localization in nodules

**Fig. 5.**
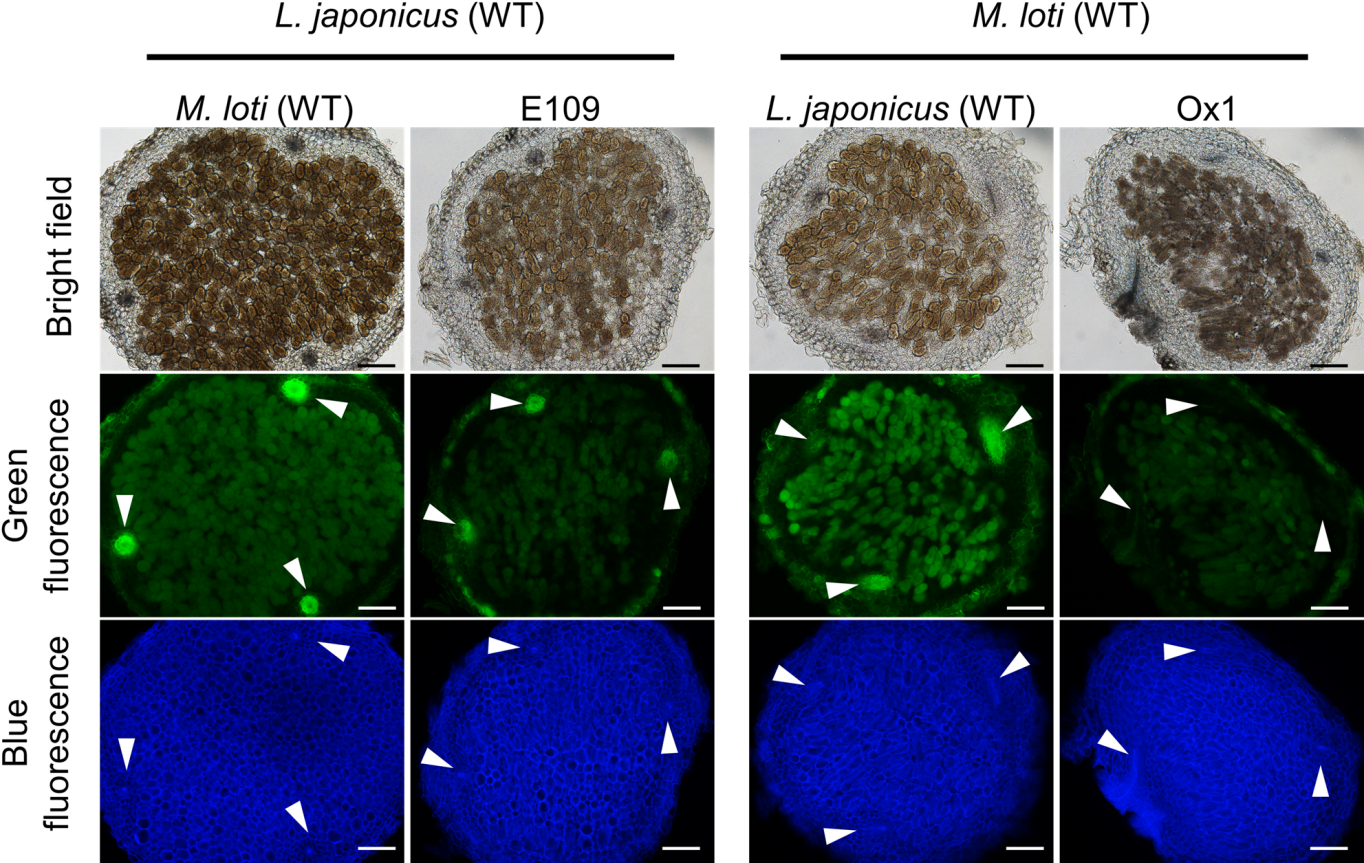
Localization of NO in nodules. Green fluorescence indicates NO labeled with DAF-FM DA. Blue fluorescence indicates nodule cell walls stained with calcofluor white. White arrowheads indicate vascular bundles in nodules. Scale bars, 100‍ ‍μm.

To investigate differences in the effects on nodules of NO detoxification by rhizobia with that by host plants, we compared these effects at 4 wpi on NO localization in the E109 *M. loti* strain, which highly expresses Hmp, with that in the Ox1 line of *L. japonicus*, which highly expresses class 1 phytogbin (Fig. 5). DAF-FM DA labeling revealed that NO-specific fluorescence intensity was lower in cells infected with E109 than in those infected with WT (Fig. 5 left-hand panel). On the other hand, when nodules in infected cells of *L. japonicus* Gifu were compared with those of the Ox1 line, they showed lower NO-specific fluorescence intensity not only in infected cells, but also in vascular bundles (Fig. 5 right-hand panel).

## Discussion

In the present study, the effects of enhanced NO-scavenging activity by rhizobia in RNS was investigated in *L. japonicus*–*M. loti* symbiosis. Enhanced NO detoxification, which was achieved by inserting the *hmp* gene from *S. meliloti* into *M. loti*, delayed the infection of *L. japonicus*. The nodules that formed after infection by the strain with *hmp* expression (E109) exhibited enhanced nitrogenase activity, but not delayed senescence. Furthermore, NO localization differed when it was scavenged by rhizobia in nodules from when it was scavenged by the host plant.

Enhanced NO detoxification by *M. loti* had a negative impact on the early stages of infection, suggesting that a certain amount of local NO is required for infection (Fig. 2a and b). The present results are consistent with previous findings; in the symbiosis between *M. truncatula* and *S. meliloti*, delayed infection was reported for high *hmp* expression in *S. meliloti* and for ectopic *hmp* expression and high *Glb1.1* expression in the hairy roots of *M. truncatula* (del Giudice *et al.*, 2011; Berger *et al.*, 2020). Although the function of NO during IT progression remains unclear, it may be closely involved in the establishment of infection. Infection in *L. japonicus* was not delayed in *LjGlb1-1* lines or hairy roots with high expression, even though root NO levels were low (Shimoda *et al.*, 2009; Fukudome *et al.*, 2019a). The rate of infection of *L. japonicus* by *M. loti* may vary depending on whether NO decreases in the whole root or at the site of infection. Although these possibilities may be examined by observing the amount and localization of NO during the formation of ITs over time, an appropriate method is not yet available. Investigations on the function of NO in the infection process will require novel methods for observing NO microscopically.

In *L. japonicus* and *M. truncatula*, the high expression of Phytogb1 in the host plant increased nitrogenase activity, while low expression decreased this activity (Shimoda *et al.*, 2009; Fukudome *et al.*, 2016; 2019a; Berger *et al.*, 2020). Nitrogenase activity was also reduced in a *hmp*-deficient strain of *S. meliloti* (Meilhoc *et al.*, 2010; Cam *et al.*, 2012). The present results demonstrated that enhanced NO removal in nodules contributed to their high nitrogenase activity (Fig. 3) and are, thus, consistent with previous findings. Other studies on the nodules of *M. truncatula* and *Glycine max* reported that NO inhibited not only nitrogenase, but also the expression and activity of leghemoglobin and glutamine synthetase (Melo *et al.*, 2011; Navascués *et al.*, 2012; Berger *et al.*, 2020), which are essential for symbiosis. Furthermore, NO repressed the expression of *nifH* and *nifD* in soybean symbiosis (Sánchez *et al.*, 2010). The expression and activity of these symbiosis-related genes and molecules need to examined in nodules induced by Hmp-expressing *M. loti*.

We previously reported that the high expression of *LjGlb1-1* delayed nodule senescence in *L. japonicus* by enhancing NO scavenging activity (Fukudome *et al.*, 2019a). Additionally, the deletion or high expression of the *hmp* gene in *S. meliloti* induced early or delayed senescence, respectively, in the nodules that formed in *M. truncatula* (Cam *et al.*, 2012). These findings suggest that the regulation of NO concentrations in nodules delays the onset of nodule senescence. In contrast, in the present study, the ectopic expression of *hmp* in *M. loti* did not delay nodule senescence (Fig. 4 and Supplementary Fig. S4). We currently cannot provide any explanations for this discrepancy. We speculate that the excessive supply of photosynthetic products by host plants to mature nodules with high nitrogenase activity may have limited the nutrient supply to old nodules. NO in nodules has also been suggested to play a role in ATP regeneration via phytoglobin–NO respiration (Igamberdiev and Hill, 2004); therefore, we cannot exclude the possibility that the excessive removal of NO may negatively affect SNF. Limited information is currently available on how much NO is required to delay senescence in old nodules, and, thus, further studies are warranted. For example, since the NO levels detected in the old nodules of WT and E109 were similar (Fig. 4b, c, and d), the rates of NO production and removal in old nodules need to be compared. The delay in nodule senescence in the Ox1 line, an overexpression line of *LjGlb1-1*, may be due to the less excessive accumulation of NO in vascular bundles. Vascular bundles of nodules are a major pathway connecting host and symbiotic organs and may function as a site for nutrient exchange and signaling (Vadez *et al.*, 2000; King and Purcell, 2005; Sulieman *et al.*, 2010; Sinclair and Nogueira, 2018; Livingston *et al.*, 2019). *LjGlb1‑1* mRNAs are mainly localized in the infected zone and in vascular bundles (Bustos‐Sanmamed *et al.*, 2011). In soybean, NO is localized in the nodule parenchyma (Calvo-Begueria *et al.*, 2018). Further studies are needed on the effects on RNS of NO localized outside of infected cells.

In RNS, the NO regulatory system is complex because NO functions either positively or negatively depending on the growth stage and location (del Giudice *et al.*, 2011; Hichri *et al.*, 2015; Fukudome *et al.*, 2016; Berger *et al.*, 2020). The genes associated with NO metabolism in bacteria are not highly conserved, which suggests that each rhizobium strain may have established its own NO metabolic pathway. The present study shows the effects of enhanced NO detoxification by *M. loti* on RNS in *L. japonicus*, which broadens our knowledge on the role that NO regulation plays in the RNS of microsymbionts and their host plants.

## Citation

Fukudome, M., Shimokawa, Y., Hashimoto, S., Maesako, Y., Uchi-Fukudome, N., Niihara, K., et al. (2021) Nitric Oxide Detoxification by *Mesorhizobium loti* Affects Root Nodule Symbiosis with *Lotus japonicus*. *Microbes Environ ***36**: ME21038.

https://doi.org/10.1264/jsme2.ME21038

## Supplementary Material

Supplementary Material

## Figures and Tables

**Table 1.** Strains and plasmids used in the present study

**Strains**	**Relevant characteristics**	**References**
*Mesorhizobium loti* MAFF303099	Wild type	Kaneko *et al.*, 2000
*M. loti*-DsRed	DsRed-labeled *M. loti* MAFF303099	Maekawa *et al.*, 2009
W315	*hmp* expression derivative from MAFF303099, plasmid (pK18mobsacB/*mlr8031*-UP/*hmp*-Gm^r^/*mlr8031*-DW) was integrated into the chromosome by homologous recombination, Km^r^, Gm^r^	This study
RW45	*hmp* expression derivative from *M. loti*-DsRed, plasmid (pK18mobsacB/*mlr8031*-UP/*hmp*-Gm^r^/*mlr8031*-DW) was integrated into the chromosome by homologous recombination, Km^r^, Gm^r^	This study
E109	*hmp* expression derivative from W315, *mlr8031*::*hmp-Gm*, Gm^r^	This study
*Escherichia coli* HST08	Cloning host	Takara Bio
**Plasmids**		
pBBR-*hmp*	Cloning vector pBBR1MCS-5 carrying *hmp*, Gm^r^	Meilhoc *et al.*, 2010
pK18mobsacB	*SacB* counterselection vector, Km^r^	Schäfer *et al.*, 1994
pK18mobsacB/*mlr8031*-UP/*hmp*-Gm^r^/ *mlr8031*-DW	pK18mobsacB carrying *hmp*, gentamicin resistance gene, and *mlr8031* flanking region; Km^r^, Gm^r^	This study
pRK2013	Co1E1 replicon carrying RK2 transfer genes, Km^r^	Figurski and Helinski, 1979
