# Supplementary material for: Nitric Oxide Detoxification by Mesorhizobium loti Affects Root Nodule Symbiosis with Lotus japonicus
Source: Microbes Environ. 2021 Aug 31;36(3):ME21038. doi: 10.1264/jsme2.ME21038 (PMC8446750; doi:10.1264/jsme2.ME21038)
Supplement: Supplementary file 1 — Supplementary Material [file 36_21038_s1.pdf]

Supplemental Figure S1

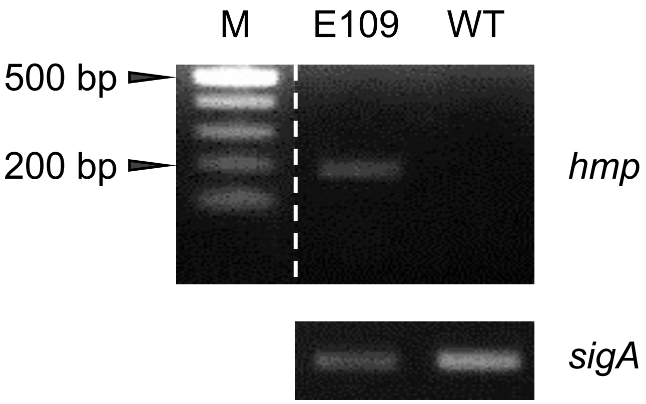

Fig. S1. Agarose gel electrophoresis of *hmp* transcripts by RT-PCR. Fragments of *hmp* and *sigA* were amplified by RT-PCR and the products were analyzed by agarose gel electrophoresis. A fragment of *hmp* was detected only in E109, whereas a fragment of *sigA* was detected in both E109 and WT. M, size marker.

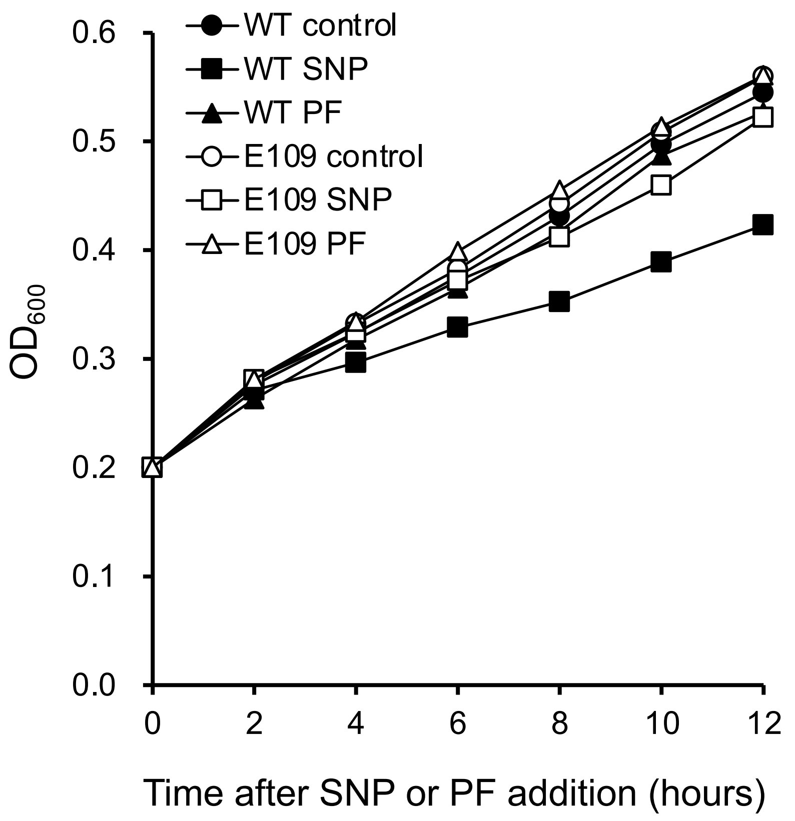

Fig. S2. Effect of PF on the growth of WT and E109. *M. loti* WT cells (black symbols) or E109 cells (open symbols) were cultured exponentially under conditions noted in the key. Cell growth was measured every 2 h after the addition of the chemical. Values are the mean of four biological replicates.

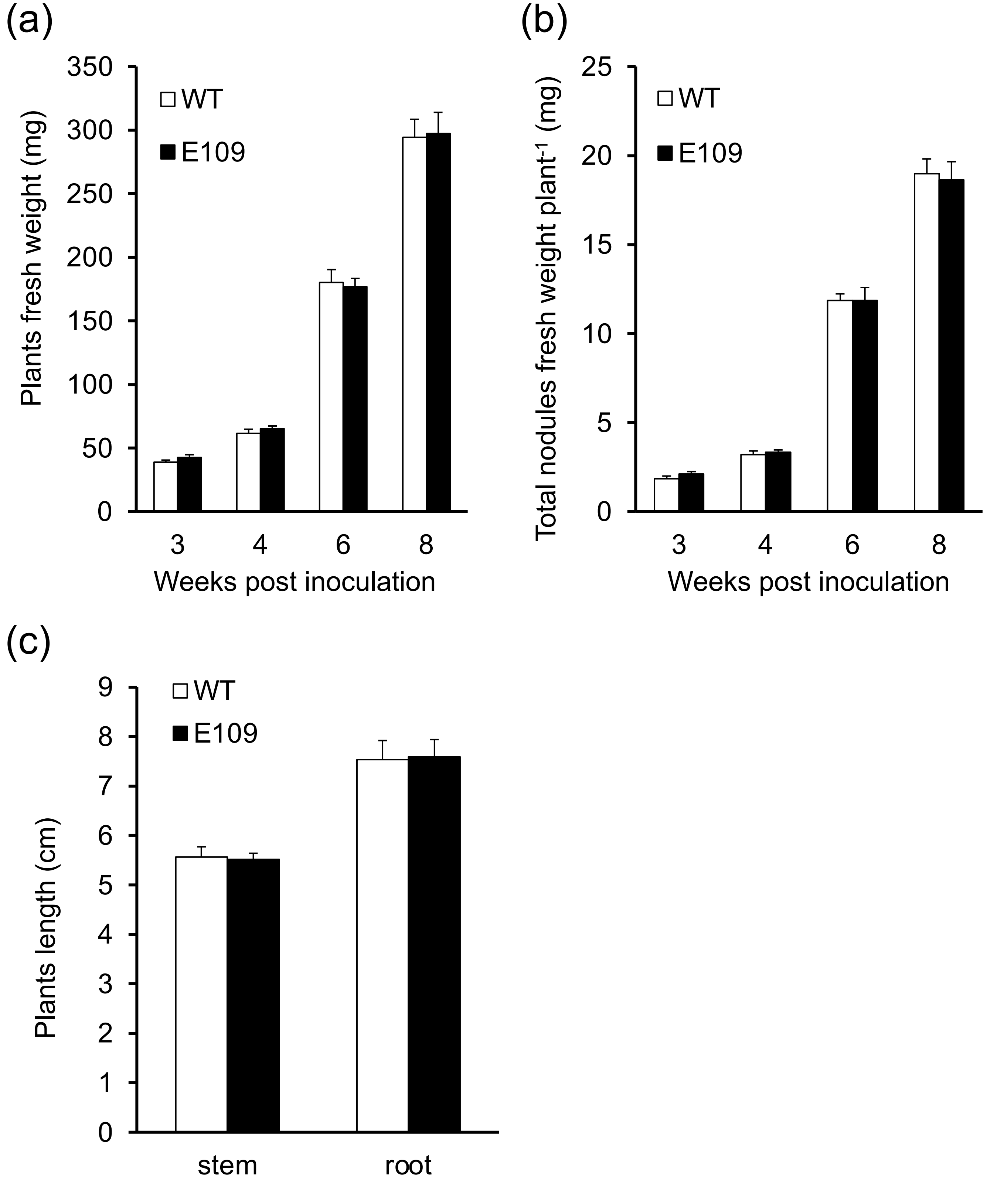

Fig. S3. Plants growth during root nodule symbiosis. The fresh weights of (a) plants and (b) nodules at 3–8 wpi, and (c) Plant length at 4 wpi. Values indicate the mean ± SE (n = 40). None of the values showed significant differences (Student's *t*-test, $P < 0.05$).

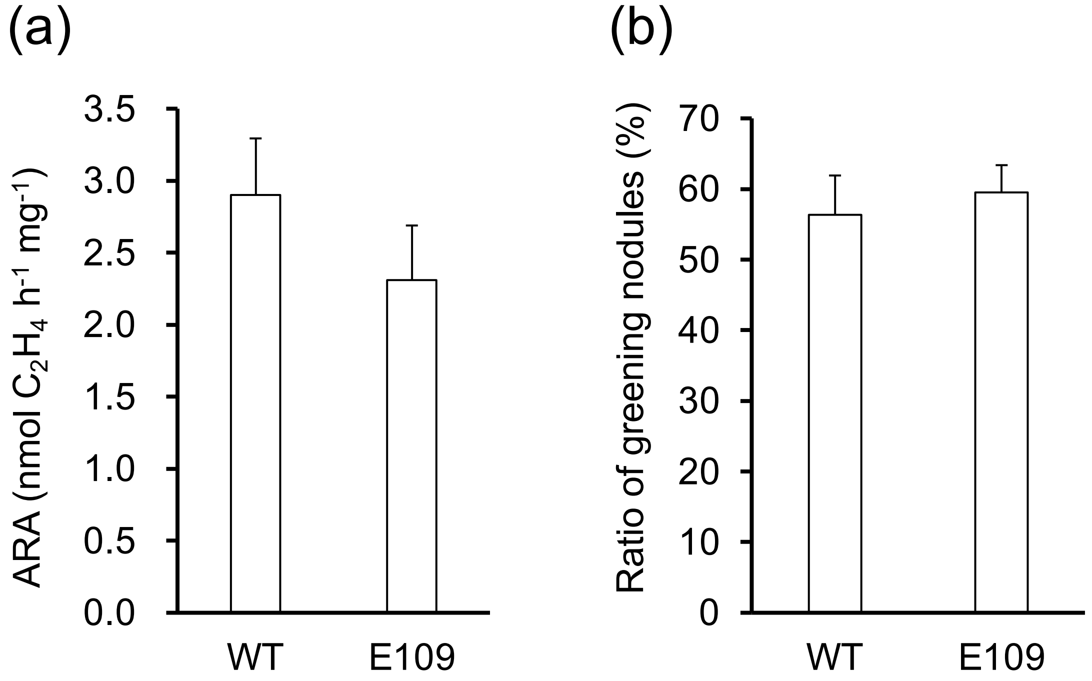

Fig. S4. Nitrogenase activity and ratio of greening in old nodules. (a) ARA was measured at 6 wpi (4 w after nodulation) and expressed as ethylene produced per h and per weight of fresh nodules. Values indicate the mean ± SE (n = 12). (b) The ratio of green nodules was measured at 6 wpi (4 w after nodulation). None of the values showed significant difference (Student's t-test, $P < 0.05$).
